# Pharmacological inactivation does not support a unique causal role for intraparietal sulcus in the discrimination of visual number

**DOI:** 10.1371/journal.pone.0188820

**Published:** 2017-12-14

**Authors:** Nicholas K. DeWind, Jiyun Peng, Andrew Luo, Elizabeth M. Brannon, Michael L. Platt

**Affiliations:** 1 Psychology Department, University of Pennsylvania, Philadelphia, Pennsylvania, United States of America; 2 Neurology Department, Mayo Clinic, Rochester, Minnesota, United States of America; 3 Department of Biology, Duke University, Durham, North Carolina, United States of America; 4 Department of Neuroscience, Perelman School of Medicine, University of Pennsylvania, Philadelphia, Pennsylvania, United States of America; 5 Marketing Department, the Wharton School, University of Pennsylvania, Philadelphia, Pennsylvania, United States of America; Centre de neuroscience cognitive, FRANCE

## Abstract

The “number sense” describes the intuitive ability to quantify without counting. Single neuron recordings in non-human primates and functional imaging in humans suggest the intraparietal sulcus is an important neuroanatomical locus of numerical estimation. Other lines of inquiry implicate the IPS in numerous other functions, including attention and decision making. Here we provide a direct test of whether IPS has functional specificity for numerosity judgments. We used muscimol to reversibly and independently inactivate the ventral and lateral intraparietal areas in two monkeys performing a numerical discrimination task and a color discrimination task, roughly equilibrated for difficulty. Inactivation of either area caused parallel impairments in both tasks and no evidence of a selective deficit in numerical processing. These findings do not support a causal role for the IPS in numerical discrimination, except insofar as it also has a role in the discrimination of color. We discuss our findings in light of several alternative hypotheses of IPS function, including a role in orienting responses, a general cognitive role in attention and decision making processes and a more specific role in ordinal comparison that encompasses both number and color judgments.

## Introduction

The “number sense” describes our ability to quantify sets without verbally counting them [1]. Educated adult humans share this number sense with infants [2,3], adults from cultures that do not have verbal counting systems [4], and other vertebrates [5,6] including rhesus monkeys [7]. It is theorized that the number sense may scaffold development of the more sophisticated concept of exact number through education [8,9], and there is evidence that the number sense continues to shape mathematical ability throughout the lifespan [10–12].

In humans, functional magnetic resonance imaging (fMRI) has revealed that activity in the horizontal segment of the intraparietal sulcus (IPS) varies with the number of items presented in a visual array [13–15] and responds to both number words and written numerals [16,17]. Children with dyscalculia, a developmental disorder of mathematical reasoning, have thinner cortical gray matter in IPS compared to children with typical calculation abilities [18,19]. Naturally occurring lesions to the IPS result in specific mathematical deficits, a condition known as acalculia [20]. Several studies have demonstrated impairment of arithmetic following disruption of IPS and neighboring regions with transcranial magnetic stimulation (TMS) [21]. Based on these observations and many others, IPS has been theorized to subserve the representation of approximate numerical magnitudes [22].

Supporting this theory, single cell recordings in monkeys have revealed neurons tuned to cardinal number in the fundus of the IPS, an area known as the ventral intraparietal area (VIP) [23–27]. These cells fire maximally to a preferred number of visually presented items and less to numbers successively more distant from the preferred. Roitman et al. [28] recorded from the lateral bank of the IPS (lateral intraparietal area, LIP) and found neurons with monotonic tuning for number rather than a preferred number. Computational models posit that the perception and discrimination of visual number passes through multiple stages. First visual stimuli are represented by units that monotonically vary activation with number, like those found in LIP. Further feed-forward processing of the monotonic signal yields a population of cardinally tuned number units like those observed in VIP [29,30]. Thus, LIP may represent an earlier stage of number processing.

It is clear, however, that the IPS contributes to many other cognitive functions as well. A handful of studies has shown that repetitive transcranial magnetic stimulation (rTMS) to parietal cortex affects the judgment of number, but also affects the judgment of non-numerical quantities such as line length, and affects the perceptual relatedness of number and non-numerical quantities [31–35]. A very large literature has demonstrated the importance of IPS, and LIP in particular, in visuospatial attention and decision making [36–38]. These studies endorse a much more general role for IPS in visual perception, attention and decision making.

Here we directly test the hypothesis that VIP and LIP play a unique causal role in numerical perception and comparison over and above any more general role they might play in ordinal comparison, in allocation of spatial attention or in perceptual decision making. We reversibly inactivated large strips of cortex comprising both regions with intracranial injections of muscimol, a GABA-a agonist that suppresses neuronal spiking activity. We assessed the monkeys’ performance on both number and color discrimination tasks employing similar stimuli, requiring identical oculomotor responses, and roughly equilibrated for difficulty. We reasoned that if LIP and VIP were even partially specialized for the processing of visual number, then inactivation of either area would result in a greater deficit in the number task than the color task. However, if inactivation resulted it similar deficits in the two tasks it would endorse the view that number processing in LIP and VIP is merely one application of a general purpose circuit for ordinal comparison, visual attention, or perceptual decision making. Our findings strongly endorse the latter hypothesis.

## Methods

### General procedures and behavioral task

All procedures were approved by the Duke University Institutional Animal Care and Use Committee. Two male rhesus monkeys (*Macaca mulatta*; 13kg and 19kg) were each implanted with a titanium head-restraint prosthesis and a neurophysiological recording chamber (Crist Instruments) over parietal cortex. Post-surgical MRI scans were used to identify prospective injection sites, which were confirmed by single cell and multi-unit electrophysiological recordings. Recordings were made using a tungsten microelectrode (FHC). Juice rewards were delivered via a tube placed in front of the monkey’s mouth and automatically controlled by a solenoid valve. Eye positions were measured (+/- 0.25°–0.5°) with an EyeLink 1000 system (SR Research) for online task control. Task software was run under MATLAB (MathWorks Inc.) using Psychophysics Toolbox 3 (http://psychtoolbox.org). Stimuli were displayed on a color LCD monitor (1,280 × 800 resolution or 1,280 x 1024, 45–55 cm viewing distance depending on the behavioral rig).

### Surgical procedures and post-operative care

All procedures were in compliance with the “Guide for Care and Use of Laboratory Animals” [39] and approved by the Duke University Institutional Animal Care and Use Committee. Animals were surgically prepared for study using standard aseptic techniques, including a head restraint prosthesis and an access port over a craniotomy through which muscimol could be administered [40]. The restraint prosthesis and access port were either implanted together in one procedure or separately in two procedures. These techniques are standard throughout the field [41–45].

Prior to surgery, each animal was sedated with a combination of intramuscular ketamine/ dexdomitor. Animals were also given intramuscular antibiotic (Baytril) prophylactically and intramuscular ketorolac as an anti-inflammatory prior to the procedure. The implantation site was shaved and pre-cleaned at this time. To sedate the animal sufficiently for intubation (as determined by corneal blink reflex and pedal reflex), the animal was administered isoflurane gas using a mask. The animal was then intubated and placed on isoflurane inhalant anesthesia through the endotracheal tube. Heart rate, blood oxygenation, ECG, temperature and respiration rate were continuously monitored to ensure safe and adequate anesthetization. Replacement fluids for insensible loss were provided (Normosol-R, 0.9% saline solution, or similar) at 5–10 ml/kg/hr. Body heat was maintained with a circulating hot water blanket. The animal was placed sternally in a stereotaxic instrument (David Kopf Instruments). Surgeons (typically 2) scrubbed and gowned while a third person, administering anesthesia, monitored the animal. The surgical site was scrubbed at least 3 times with a betadine or chlorhexidine disinfectant alternating with 70% isopropanol. The surgeons then draped the sterile field.

A portion of the dorsal scalp was removed and the skin bordering the incision was retracted. A small craniotomy was then made using a sterile drill and bits. Sterile surgical-grade titanium bone screws were then implanted into the skull. A stereotaxic device was used to lower the sterile access port (Crist Instruments) over the craniotomy. A sterile titanium head restraint post (Crist Instruments) was also lowered stereotaxically into contact with the bone. The access port and head restraint post were then bonded to the screws with sterile bone cement. The interior of the access port was then lavaged with sterile saline and antibiotic (Baytril), and closed with a sterile cap to prevent contamination. The site was checked for tight opposition to the cranial implant. Ketamine/ dexdomitor was reversed with intramuscular antisedan upon completion of the procedure.

After surgery animals were continuously monitored for at least three hours and until sternal recumbency was regained and maintained. Buprenorphine (0.01 mg/kg) was administered at this time and again every 8 hours as prophylactic pain management for 72 hours following the procedure. Observations were made at least as frequently as the analgesic schedule including at night, and animals were regularly monitored by lab members and veterinary staff during the day.

### Animal husbandry and disposition

Monkeys were obtained from the Mannheimer Foundation in Florida where they were bred and housed before being acquired by the lab and transported to Duke University. As recommended by the USDA Environmental Enhancement for Nonhuman Primates [46] and the National Research Council’s Psychological Well-Being of Nonhuman Primate [47], we provided enrichment by socially housing our monkeys when possible and by providing toys, games, and puzzles that must be manipulated to obtain food and treats hidden inside. Monkeys were housed in Primate Products cages which allow frequent access to a two-level activity module (daily access under normal circumstances), which provided extra space for socialization, movement, and larger toys.

Monkeys received their main sustenance from nutritionally balanced biscuits. They received daily supplements of fruit, nuts and seeds for enrichment. To maintain task motivation access to water was regulated prior to experimental sessions. While participating in the study, animals received a minimum 20 mL/kg of water a day with the opportunity for more during the task. When an animal was not tested for more than five days it was provided at least 50 mL/kg of water a day; during longer periods off-study animals were permitted free access to water. These amounts were expected to provide sufficient daily fluid requirement [48–50]; however, each animal on regulated access to fluid was also observed daily for health status and hydration. Daily hydration status was assessed by general appearance (bright, alert, responsive), body weight, skin turgor, and fecal output/ consistency by members of the laboratory and veterinary staff.

In order to reduce the total number of animals used in our research, where possible animals were used for more than one experiment. After being used in the present study both animals continued to be used for other behavior and neurobiological experiments. One animal is still living and actively used in research in our lab. The other died peacefully of old age.

### Behavioral tasks

We trained monkeys on two discrimination tasks, a color task and a number task. In both tasks, monkeys aligned gaze within 2.5° of a central target to initiate a trial. After 700 ms, two arrays of dots (0.20° to 0.68° in diameter) on gray circular backgrounds (4.5° in diameter) were simultaneously displayed in two of the four visual quadrants around the fixation point (center of stimulus at eccentricity 8°–12°). The stimulus arrays were displayed for 70 ms or 300 ms depending on animal. After the dot arrays extinguished, monkeys were required to wait an additional 400 ms for the fixation cross to extinguish, which served as the cue to shift gaze to the previous location of one of the two stimuli. The monkey’s choice was registered as soon as he shifted gaze into an invisible square area (4.5° each side) circumscribing the previous location of each stimulus. The trial was aborted if the monkey took longer than 1,500 ms to select a stimulus or if gaze deviated further than 12° from fixation without entering one of the stimulus choice areas. No other constraint was placed on the monkey’s gaze position, and he was free to make multiple saccades until one of these conditions was met. Response time was measured from fixation offset to the time gaze entered a target area. If the monkey selected the location of the “correct” stimulus (contingencies described for each task below), he was rewarded with ~0.15 ml juice. Abort trials or incorrect stimulus selection were followed by an audio tone (521 Hz), and a correction trial. Correction trials consisted of an exact repeat of the previous trial, with the same stimuli. These correction trials continued to repeat until the monkey responded correctly and reward was delivered. Correction trials were excluded from analysis. Each experimental session consisted of at least 2,300 intermixed color and number trials, excluding correction trials.

One stimulus array was always displayed in the upper visual hemifield and the other was always in the lower hemifield. For monkey Sh, the upper and lower arrays were placed randomly on the right or left side. For monkey Br, however, both the upper and lower arrays were always placed together on either the left or the right. We made this change after preliminary analysis of Sh’s data showed that many of the effects of muscimol injection were restricted to trials on which both stimuli were displayed in the hemifield contralateral to the injection site. We excluded trials from Sh with mixed presentation side from further analysis. We also excluded trials in which monkeys broke fixation early, in which monkeys took more than 1.5 s to shift gaze after the go-cue, or in which monkeys shifted gaze outside the target area (greater than ~12° from fixation). In Experiment 1, inappropriate responses comprised 5.5% (std. 3.6 percentage points) of saline and 5.1% (std. 2.6 percentage points) of muscimol trials; this difference was not significant (*F*(1,26) = 0.15, *p* = 0.71). In Experiment 2, they comprised 4.4% (std. 1.8 percentage points) in the saline and 4.7% (std. 2.3 percentage points) in the drug condition; this difference was not significant (*F*(1,27) = 0.17, *p* = 0.68). These percentages and standard deviations represent mean and variance across sessions.

### Number and color stimuli

The description of the behavioral tasks above applies to both the number and color tasks; below we describe the differences between the tasks. In the number task, the dots within each stimulus array were fully color saturated. Within each array, some dots were red and some were another color, either green or blue. The correct array was the one with the greater number of red dots. The incorrect array always had the same total number of dots with the inverse proportion of red dots to green or blue dots. For example, if the correct array had 11 red dots and 5 blue dots, then the incorrect array would have 11 blue dots and 5 red dots. The total number of dots in each array was always 8, 16 or 32. For 16 total dots, the ratio of red to non-red dots was 9:7, 10:6, 11:5, and 12:4. For 32 total dots the ratio was 18:14, 20:12, 21:11, and 24:8. For 8 total dots, the ratio was 6:2 and 5:3. We used different numerical ratios to modulate task difficulty for each monkey, and not all numerical pairs were shown to both monkeys. All dots ranged from 0.20° to 0.68° in diameter. For half of all trials, the total dot area (number of pixels) of each color within each array was equal, and for the other half of the trials, dot size was randomly and independently determined. This manipulation allowed us to assess the effect of total area and item area on numerical and color discrimination, and to ensure that animals did not rely on these visual features alone to make their responses.

The color stimulus array pairs were identical to the number stimuli except that all the dots within an array were the same color, but the color of the dots varied between arrays. The color was a combination of red and either green or blue. The correct stimulus was the array with greater saturation of red. The relative ratio of red and either green or blue determined trial difficulty. This combination resulted in arrays that were either yellowish (when combined with green) or purplish (when combined with blue). Monkeys were rewarded for selecting the “redder” stimulus.

### Site selection and electrophysiological recording

**Fig 1:**
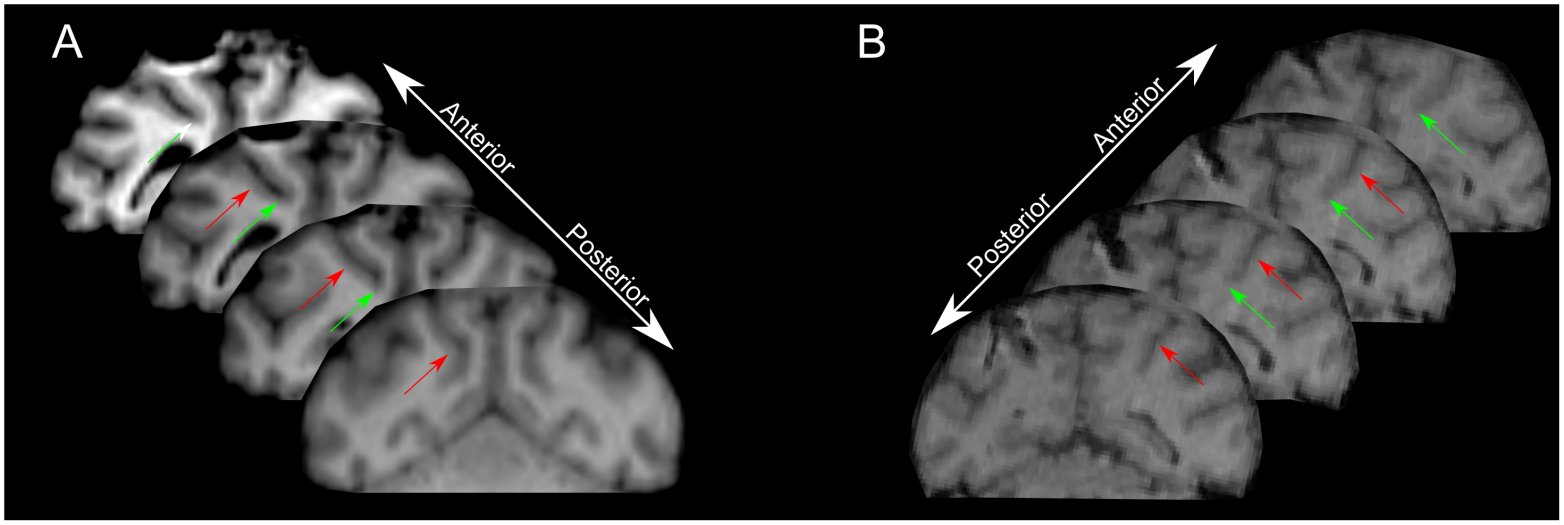
Injection site targets indicated on MR images. (A) Monkey BR and (B) monkey SH. Green arrows indicate VIP injection sites and red arrows indicate LIP injection sites.

Single unit recording sessions were conducted before muscimol or saline infusion to identify the location of LIP and VIP. Recordings were conducted using single tungsten microelectrodes (FHC, Bowdoin, ME), a dura piecing guide tube (23 gauge), and a Kopf (David Kopf Instruments, Tujunga, CA) hydraulic microdrive system. Neuronal signals were amplified and digitized using the Plexon system (Plexon, Dallas, TX). We used single-cell and multi-unit activity to confirm the predicted location of gray and white matter boundaries obtained from MRI, and to measure the exact distances on the microdrive insuring injections were made at the appropriate depth. Fig 1 shows the targeted injection locations superimposed on the MRIs from each animal.

In addition, we functionally confirmed LIP injection locations using a delayed saccade task. Monkeys fixated a central point (±2.5°) to initiate a trial, a target flashed at 4.5° or 9° eccentricity in a random direction from the central fixation point for 200 ms, followed by a 400 ms—600 ms delay. After the delay the central fixation point extinguished and monkeys shifted gaze to the previous location of the target within 300 ms. We confirmed single- and multi-unit perisaccadic activity at the putative LIP injection locations [51].

VIP injection locations were functionally confirmed with a moving dot array stimulus. A field of 290 randomly placed dots (0.55° each) filled the display screen. Monkeys fixated a central point to initiate a trial, all the dots on the field moved in one of eight directions (0°, 45°, 90°, 135°, 180°, 225°, 270°, or 315° from straight up) at one of three speeds (40°, 80°, or 120° of visual angle per second) for 300 ms. Monkeys were rewarded for maintaining fixation through the trial. As dots moved off the screen they were replaced with dots moving onto the screen from the other side. We confirmed strong single- and multi-unit activity in response to visual motion in the putative VIP injection locations [52].

### Intracranial drug administration

Muscimol (Sigma-Aldrich Co. LLC, St. Louis, MO), a GABA-a agonist, was used to transiently and unilaterally inactivate either VIP or LIP. Muscimol was dissolved in saline to a concentration of 5 mg/ ml. Saline was used as vehicle control. Three injection sites were identified for each area in each animal spaced at approximately 2 mm. At the beginning of each session the injection needle (outer diameter: 160 μm; Hamilton Co., Reno, NV) was inserted through the guide tube through the appropriate grid hole (Crist Instruments) and driven to the appropriate depth. 2 μl of muscimol solution or saline were infused at the site at a speed no greater than 1 μl /min. The needle was then withdrawn and the same procedure was repeated until injections had been completed at all three sites. It took approximately one hour to complete all three injections. The task was run for a few minutes (approximately 100 trials) for performance adaptation either before or during the injection. The task was restarted immediately after the needle was retracted from the third injection and continued until at least 2,300 trials were completed (approximately 2.5 hours).

Muscimol and saline sessions were intermixed over days following an ABBA pattern so that any change in performance over days or weeks would average out. Each experiment (LIP or VIP) consisted of 15 or 16 injection sessions in each of the two animals.

### Data analysis

Response time (RT) was defined as the time from fixation cross offset to the time that the monkeys had shifted their gaze outside the fixation window. We fit generalized linear models (GLM) to both accuracy and RT data to determine whether drug or saline administration affected task performance. We used a logistic regression (logit link function and binomial error distribution) to model accuracy and used a log link function and a gamma error distribution to model RT. The GLMs contained six fixed effects regressors for drug (muscimol or saline vehicle), task (number or color), visual hemifield of the stimuli (contralateral or ipsilateral), time within session (first half or second half), and two parametric regressors for task difficulty: numerical ratio and color saturation difference. We included a full cross of all interaction terms, except for interactions of task with difficulty, which would have over-specified the model. We also included random effects regressors for session and monkey. Table 1 gives details on the full models and S1 and S2 Tables give details on the parameter estimates. The model was fit using the Matlab statistics and machine learning toolbox (MathWorks Inc.).

**Table 1:** Model specification and descriptive statistics.

	Exp 1: VIP Acc	Exp 1: VIP RT	Exp 2: LIP Acc	Exp 2: LIP RT
*Num. of Observ.*	64978	64978	67500	67500
*Fixed Coefs.*	29	29	29	29
*Random Coefs.*	30	30	31	31
*Distribution*	Binomial	Gamma	Binomial	Gamma
*Link*	Logit	Log	Logit	Log
*AIC*	47622	-2.4e+05	49761	-2.4e+05
*BIC*	47904	-2.4e+05	50044	-2.4e+05
*Log Like.*	-23780	1.2e+05	-24849	1.2e+05
*Deviance*	47560	-2.4e+05	49699	-2.4e+05

## Results

**Fig 2:**
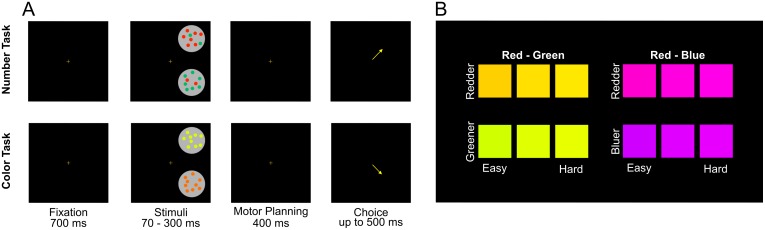
Task structure and hues used in the color task. (A) Monkeys fixated on a central stimulus. After 700 ms two stimuli appeared on the screen simultaneously, remained for 70 or 300 ms and then disappeared. The fixation-cross remained for 400 ms and then extinguished, after which the monkey could indicate a choice by shifting gaze to the location where a stimulus had been. In the number task, the stimulus with the larger number of red dots was correct. In the color task, the stimulus with dots with more red hue saturation was correct. Hue differences in the color task in panel (A) are exaggerated to make the correct answer more obvious. Correct responses were rewarded with juice. Incorrect responses resulted in a repeated trial. (B) Actual hues used for dot stimuli in the color task. Stimuli in the top row had redder hue and were correct. Each stimulus in the top row was paired with the hue below it. Red-green and red-blue trials were randomly interleaved.

Monkeys performed a number discrimination task, in which they had to choose the stimulus containing the greater number of red dots (Fig 2A), and a color discrimination task, in which they had to choose the stimulus with a redder hue (Fig 2B). These two types of trials were intermixed with each other within a session. Both monkeys performed both tasks well above chance expectations prior to injections. The difficulty of the numerosity task was modulated by the numerical ratio of the red dots to dots of another color (blue or green) in the arrays being discriminated; similarly, performance on the color task was modulated by the difference in red color saturation relative to a second color (blue or green) saturation between the stimuli. To test whether VIP or LIP plays a causal role in numerosity discrimination, we injected muscimol or saline vehicle into VIP or LIP in two separate experiments.

### Experiment 1: VIP inactivation

In Experiment 1 we unilaterally inactivated VIP in two monkeys and measured the effect on performance in the color and number discrimination tasks. We ran a global GLM on accuracy and RT. The critical test of our hypothesis would be revealed in interactions that included both drug and task or drug and task difficulty. We also considered the possibility that these effects might only manifest themselves within the visual hemifield contralateral to the injection site, or might be most pronounced in either the first or second half of the session depending on the rate of drug diffusion and the rate of biological degradation of the muscimol.

**Fig 3:**
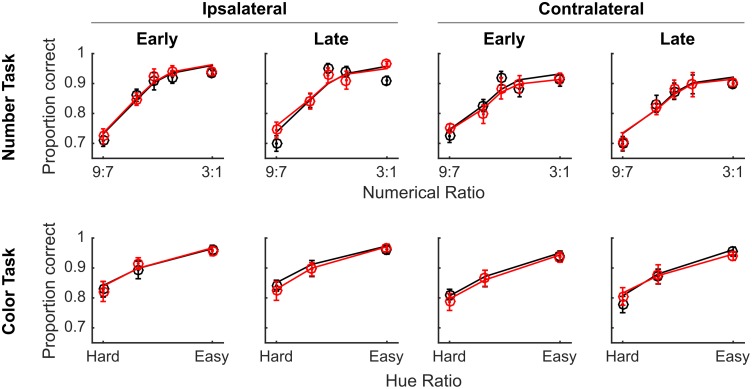
VIP inactivation did not affect accuracy on either task. Accuracy data from Experiment 1. Mean accuracies were calculated separately for the full cross of drug administration (red circles for musicmol and black circles for saline vehicle), task (number or color discrimination), side (ipsilateral or contralateral to injection site), time in session (early or late based on median split of time in session), and task difficulty along the x-axis (based on numerical ratio or relative hue saturation). Curves represent the predictions of the logistic GLM fit to the data. Data was collapsed across subject and session number, which were treated as random effects in the GLM. Error bars represent standard errors across sessions.

We found no overall main effect of muscimol on accuracy (*t*(64949) = -0.49, *p* = 0.62), and no significant interactions involving drug (all *p* > 0.1). Indeed, the full GLM failed to explain significantly more variance than a reduced model that excluded regressors for drug or interactions involving drug (*χ*^2^(16) = 14.61, *p* = 0.55), indicating that VIP muscimol injection had no discernable effect on accuracy in either task (Fig 3).

**Fig 4:**
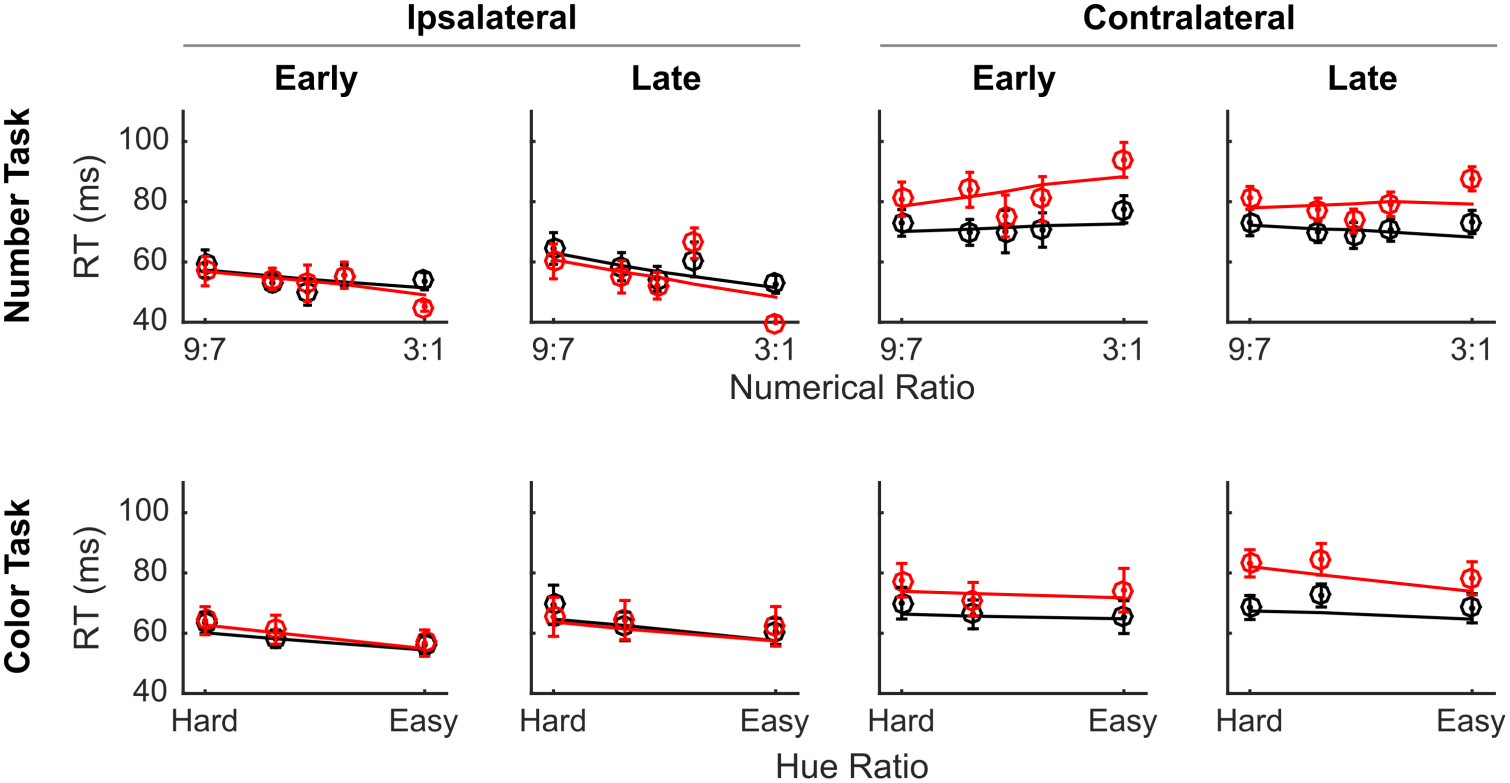
VIP inactivation slowed RTs for both numerical and color comparisons performed in the contralateral visual hemifield. The same conventions as Fig 3, but with RT on the y-axis. Red curves indicate sessions with muscimol and black curves indicate sessions with saline vehicle. Circles indicate the empirical data averages and lines show the model fit. Error bars indicate standard errors across sessions.

We examined the effect of VIP muscimol on RT using a GLM with the same regressors and found no significant main effect of drug on RT (*t*(64949) = 1.03, *p* = 0.30). However, we did find that the full model explained significantly more variance in RT than a model that excluded terms for drug and all interactions that included drug (*χ*^2^(16) = 89.35, *p* << 0.0001), providing very strong evidence that injecting muscimol into VIP affected RT. We examined these differences in more detail by breaking out the data by drug, side, time, numerical ratio or color difference (Fig 4). It is clear from visual inspection that there was an effect of muscimol on RT during trials presented in the visual hemifield contralateral to the injection site, which we confirmed by comparing the full model against a reduced model without interactions between drug and side (*χ*^2^(8) = 77.93, *p* << 0.0001).

However, we found no unique effect of muscimol on one task compared to the other. We tested our full model against a reduced model missing interactions of drug-by-task and drug-by-task-difficulty and found that there was no significant variance explained by these terms (*χ*^2^(12) = 18.27, *p* = 0.11).

**Fig 5:**
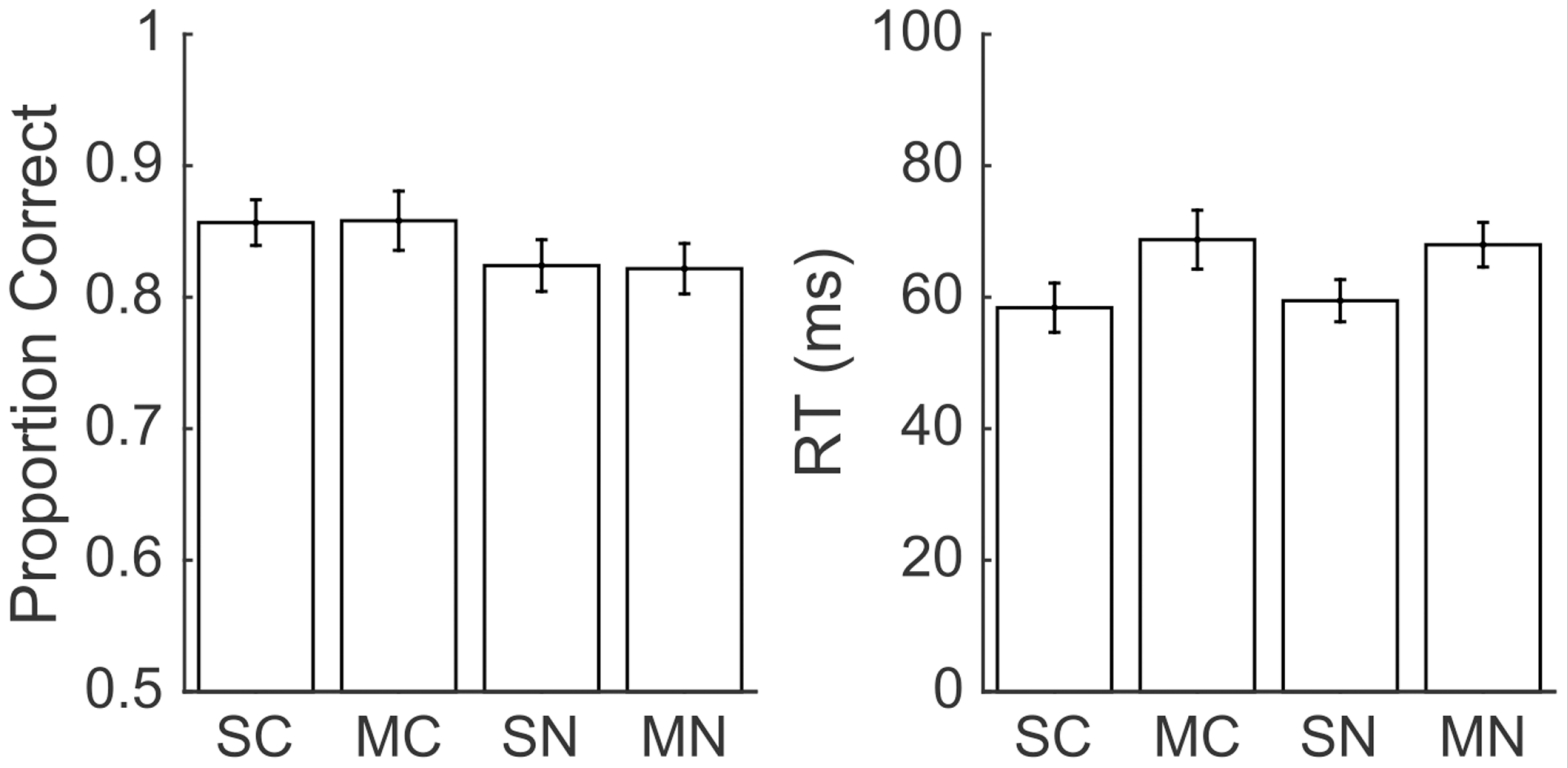
VIP muscimol injections significantly slowed RT, but there was no interaction with task. Accuracy and response time effects in Experiment 1 during contralateral late trials only. SC is saline color task, MC is muscimol color task, SN is saline number task, and MN is muscimol number task. Error bars indicate standard errors across sessions.

We found that the strongest effect of muscimol was during trials presented in the visual hemifield contralateral to the injection site, especially in the second half of the session. To confirm our findings from the GLM and to improve our power to detect an interaction between task and drug effects, we performed a post-hoc analysis on only these trials. We calculated accuracy and median RT on correct trials for each session and for each task (Table 2; Fig 5). We found that accuracy on the color task was significantly greater than on the number task (paired t(27) = 4.02, p = 0.0004), but response time was similar in both tasks (paired t(27) = -0.10, p = 0.92). Accuracy following muscimol was similar to accuracy following saline injection (t(26) = 0.01, p = 0.99), but response times were marginally slower following muscimol (t(26) = -1.79, p = 0.08). To determine whether VIP inactivation selectively disrupted numerical cognition we subtracted proportion correct on the number task from proportion correct on the color task (within session) and tested whether this difference was significantly affected by muscimol injection (across session). We found no significant effect of drug on accuracy (t(26) = -0.22, p = 0.83). Similarly, we examined the difference in median RT between the tasks and whether it was affected by muscimol, which it was not (t(26) = -0.60, p = 0.55). Thus, we confirmed the finding from the GLM that although VIP muscimol injections slowed response times to targets in the contralateral visual field, this effect was uniform across both tasks.

**Table 2:** Accuracy and RT data from Experiment 1 (VIP) trials presented contralateral to injection in the second half of the experimental session.

	Proportion Correct	RT
Color Task (all sessions)	0.86 (0.07)	64 (16) ms
Number Task (all sessions)	0.82 (0.07)	64 (13) ms
Saline Sessions	0.84 (0.07)	59 (12) ms
Muscimol Sessions	0.84 (0.08)	68 (14) ms
	Proportion Correct (Task Difference)	RT (Task Difference)
Saline Sessions	0.03 (0.04)	-1.1 (8) ms
Muscimol Sessions	0.04 (0.05)	-0.8 (8) ms

Values indicate means across sessions with standard deviations in parenthesis. RT values are means and standard deviations of the median session RT (correct trials only). The bottom two rows indicate the means and standard deviations (calculated across sessions) of the difference between the color and number tasks (calculated within each session). Values were calculated by subtracting number task values from color task values.

### Experiment 2: LIP inactivation

In Experiment 2 we unilaterally inactivated LIP in the same two monkeys and measured the effects on performance in the same color and number discrimination tasks. The data were subjected to the same global GLMs for accuracy and RT.

**Fig 6:**
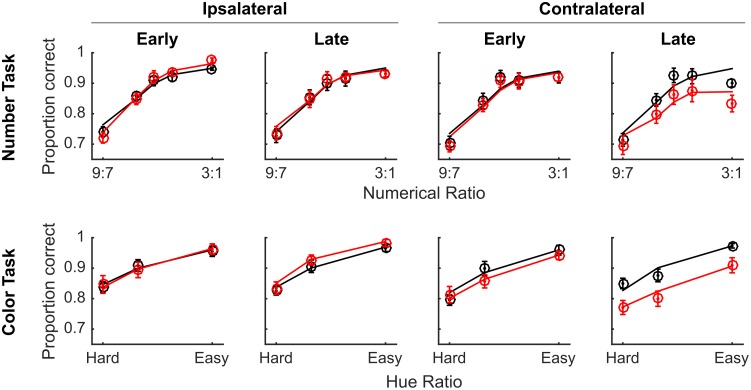
LIP inactivation impaired discrimination performance of stimuli in the contralateral visual hemifield for both color and numerical comparisons. The same conventions as Fig 3 apply. Red curves indicate sessions with muscimol and black curves indicate sessions with saline vehicle. Circles indicate the empirical data averages and lines show the model fit. Error bars indicate standard errors across sessions.

To examine the effects of muscimol on accuracy in detail, we broke out the accuracy data by side, time, task, drug, and difficulty (Fig 6). LIP inactivation did not result in a significant decrease in overall accuracy (*t*(67471) = -1.63, *p* = 0.10), however, the full model explained significantly more variance than a reduced model missing terms for drug and all interactions with drug (*χ*^2^(16) = 130.4, *p* << 0.0001), providing strong evidence of some effect of LIP muscimol on accuracy. The most obvious effect is the decrement in performance on contralateral trials late in the session. There was a significant effect of muscimol on the shape of the difficulty curve in both tasks demonstrated by significant interaction terms for drug-by-time-by-numerical-difficulty (t(67471) = -3.74, *p* = 0.0002) and drug-by-side-by-time-by-color-difficulty (t(67471) = -2.73, *p* = 0.006). However, muscimol affected the two tasks similarly: there was no significant interaction that included drug and task type (all *ps* > 0.1), and the full model did not explain significantly more variance than a model missing all interactions of drug and task type (*χ*^2^(4) = 4.03, *p* = 0.41).

**Fig 7:**
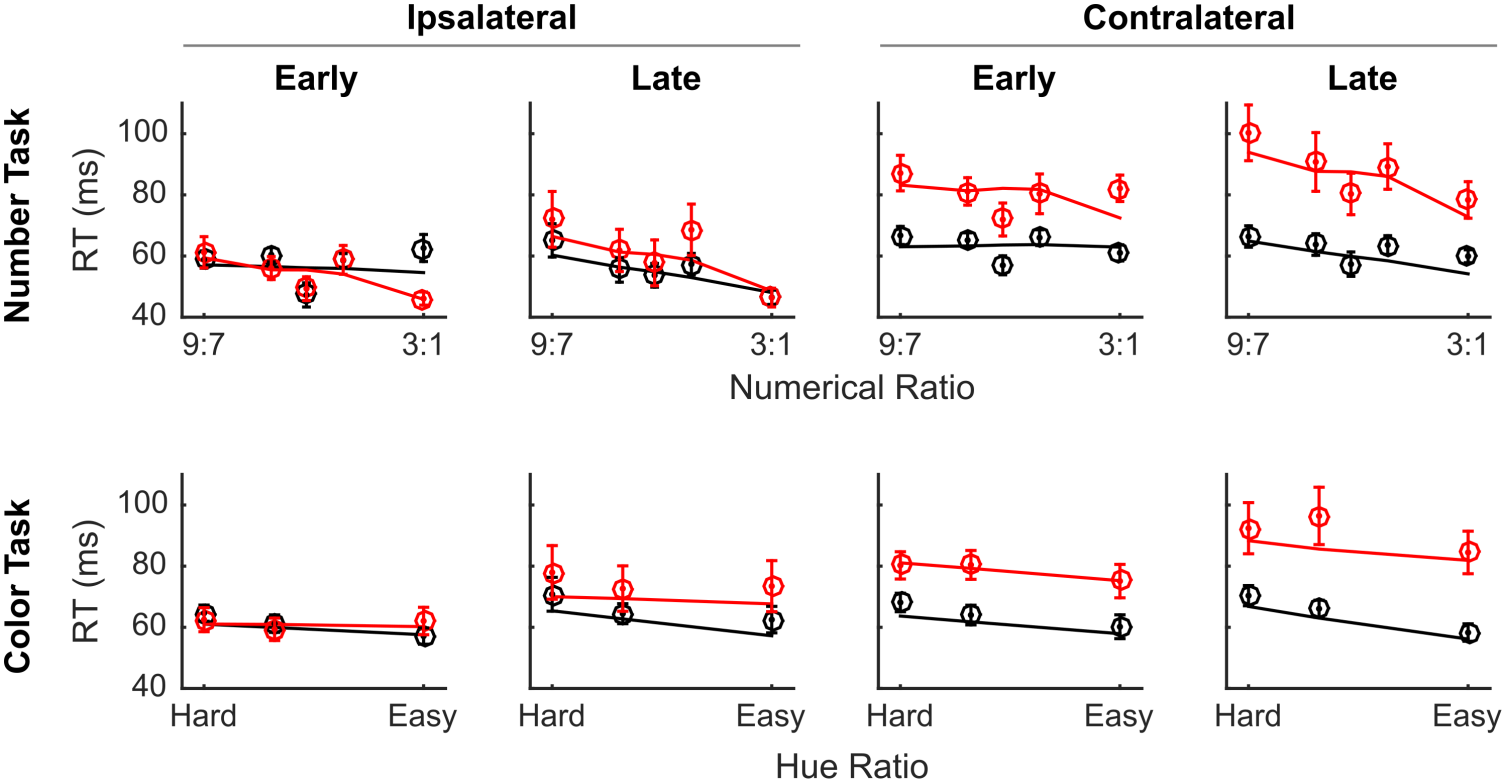
LIP inactivation slowed RT for both numerical and color comparisons made in the contralateral visual hemifield. On average, drug induced slowing was slightly greater on number than on color trials. Red curves indicate sessions with muscimol and black curves indicate sessions with saline vehicle. Circles indicate the empirical data averages and lines show the model fit. Error bars indicate standard errors across sessions.

There was no significant main effect of LIP muscimol on RT (*t*(67471) = 1.67, *p* = 0.10), however, there were significant interactions between drug and task variables including drug-by-task (*t*(67471) = 1.95, *p* = 0.05), drug-by-numerical-difficulty (*t*(67471) = -2.15, *p* = 0.03), and drug-by-time-by-numerical-difficulty (*t*(67471) = 2.01, *p* = 0.04). Fig 7 shows the LIP RT data broken out by side, time, drug and difficulty. Again, the effects of muscimol were most pronounced on trials presented in the contralateral visual hemifield, especially later in the session.

**Fig 8:**
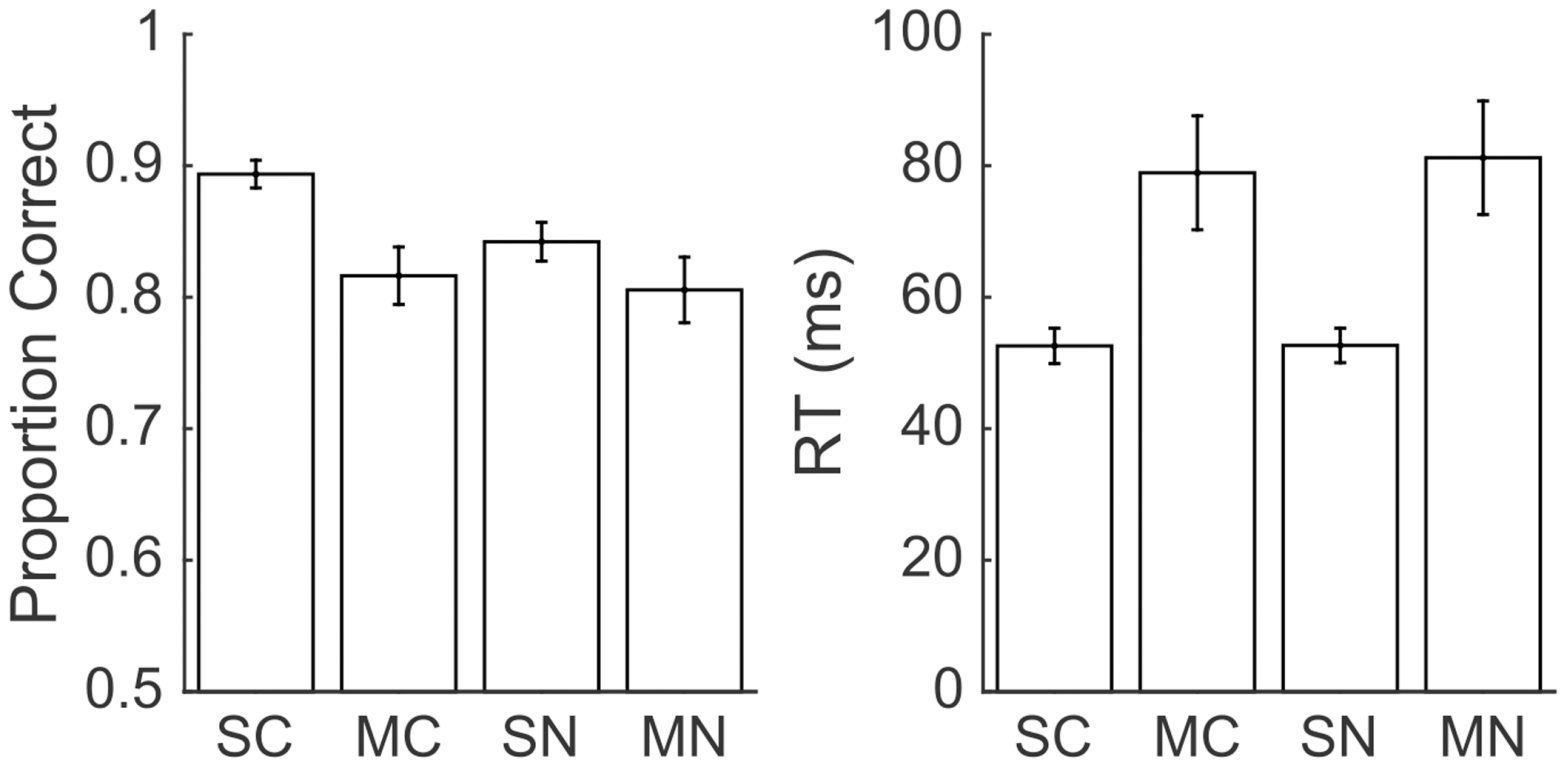
LIP inactivation caused a significant deficit to accuracy and slowing of RT in both tasks. Accuracy and response time effects in Experiment 2 during contralateral late trials only. SC is saline color task, MC is muscimol color task, SN is saline number task, and MN is muscimol number task. Error bars indicate standard errors across sessions.

To confirm our findings from the GLM and to improve our power to detect an interaction between task and drug effects, we performed the same post-hoc tests as in Experiment 1 (Table 3; Fig 8). We found that accuracy was significantly higher in the color task than the number task (paired t(28) = 3.21, p = 0.0034), but that RT was similar (paired t(28) = -1.01, p = 0.32). Following muscimol we found a significant impairment in accuracy (t(27) = 2.08, p = 0.05) and a significant slowing of RT (t(27) = -3.20, p = 0.004). We also observed a small but significant interaction between muscimol and task on accuracy (t(27) = 2.18, p = 0.04). However, the effect was the opposite of expected; the color task was more impaired by muscimol than the number task. There was no interaction effect on RT (t(27) = 0.98, p = 0.33).

**Table 3:** Accuracy and RT data from Experiment 2 (LIP) trials presented contralateral to injection in the second half of the experimental session.

	Proportion Correct	RT
Color Task	0.86 (0.07)	65 (27) ms
Number Task	0.82 (0.08)	66 (27) ms
Saline Sessions	0.86 (0.05)	52 (10) ms
Muscimol Sessions	0.81 (0.09)	80 (32) ms
	Proportion Correct (Task Difference)	RT (Task Difference)
Saline Sessions	0.05 (0.05)	-0.1 (5) ms
Muscimol Sessions	0.01 (0.05)	-2.3 (7) ms

Values indicate means across sessions with standard deviations in parenthesis. RT values are means and standard deviations of the median session RT (correct trials only). The bottom two rows indicate the means and standard deviations (calculated across sessions) of the difference between the color and number tasks (calculated within each session). Values were calculated by subtracting number task values from color task values.

In summary, LIP inactivation caused a decrement in accuracy and a slowing of RT on trials presented contralateral to the injection sites towards the end of the sessions. The GLM approach indicated that an interaction between task and drug treatment on RT, but our post-hoc tests failed to detect it. The post-hoc test did reveal a small but significant interaction between task and drug treatment on accuracy, which was absent from the GLM. We found a decrement in accuracy that was slightly greater in the color task. During saline control, performance on the color task was 5 percentage points better than on the number task, after muscimol the performance was only 1 percentage point better on the color than on the number task.

### Demonstrating number and color discrimination did not rely on area

Prior studies have shown that monkeys readily perform numerical discriminations without relying on non-numerical cues such as area [7,24]. Nevertheless, we analyzed the behavioral data to ensure that animals were utilizing number and color in the two respective tasks. In the number task, half of trials controlled for the total area covered by the dots of each color so that it did not vary with their number, and the other half of trials controlled individual item area so that it did not vary with number.

**Fig 9:**
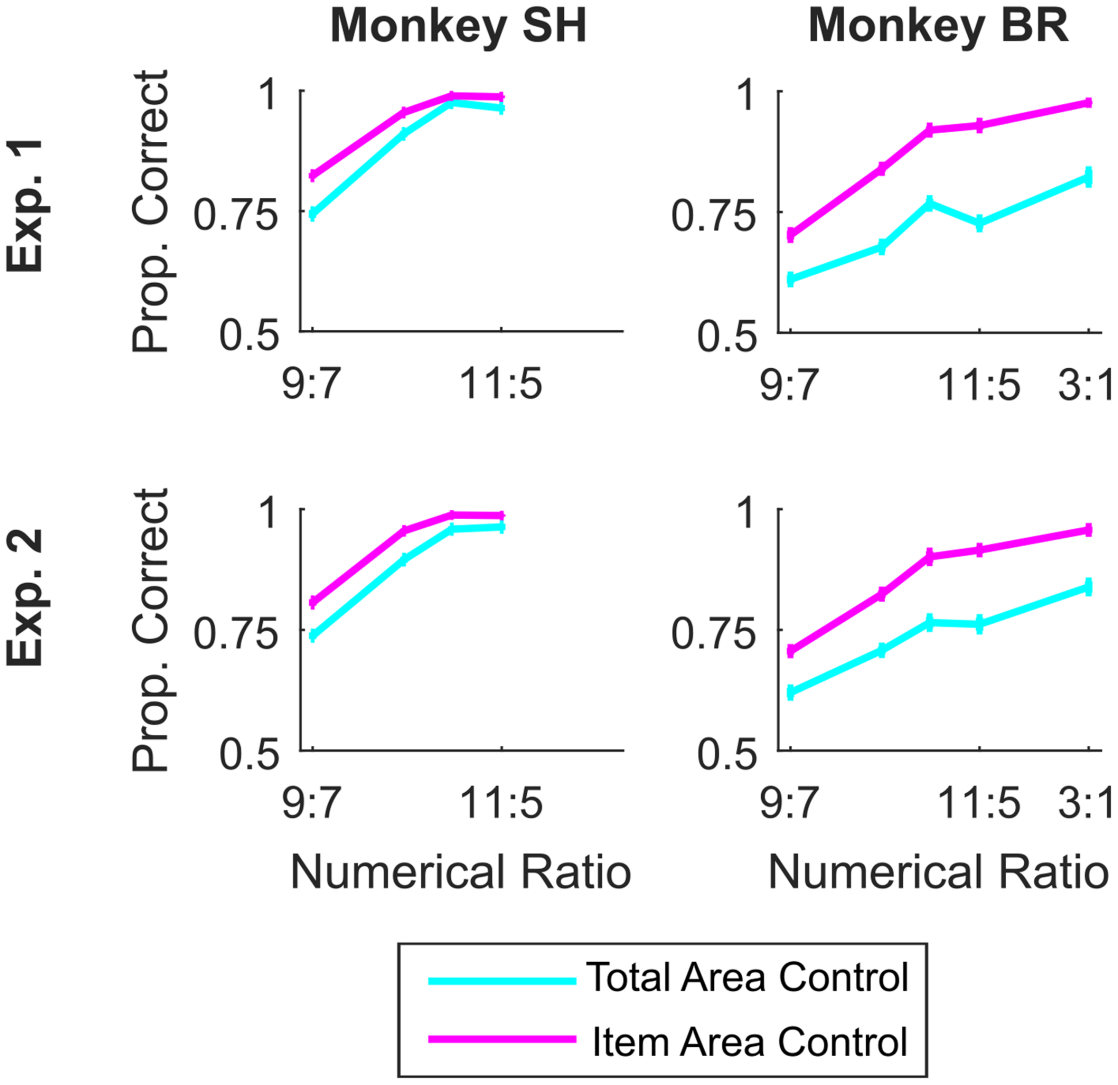
Performance in the number task was lower when total area was controlled, but numerical ratio effects were observed in both stimulus control conditions. Accuracy over numerical ratio for the number task in Experiments 1 (top row) and 2 (bottom row) separated by animal (monkey SH in the left column and monkey BR in the right column). Cyan shows accuracy in the total area control condition, and magenta shows accuracy in the item area control condition. Error bars indicate standard error calculated across sessions.

In Experiment 1, mean proportion correct across sessions was significantly lower (paired t(27) = 10.31, p << 0.0001) when total area was controlled (M = 0.78, SD = 0.08) than when item area was controlled (M = 0.88, SD = 0.04) indicating that the perceptual discrimination was easier when area and number were congruent, as has been observed in humans [53,54]. However, in both cases performance was well above chance (lowest t(27) = 17.48, highest p << 0.0001), demonstrating that area could not have been the only basis for discrimination. We also found that there was a significant parametric effect of numerical ratio when total area was controlled (t(124) = 4.40, p << 0.0001) and when item area was controlled (t(124) = 9.11, p << 0.0001), indicating that number played a role in stimulus selection in both trial types (Fig 9). The color task could not be performed above chance by relying on total area, because area and hue were uncorrelated, and so the finding from the GLM that color performance was above chance and that it depended on hue in Experiment 1 confirmed that performance did not depend on area. Nevertheless, we did find a small but significant effect of area on color performance. A median split of total area showed that proportion correct was significantly greater (paired t(27) = 3.79, p = 0.0008) when there were more illuminated pixels (M = 0.89, SD = 0.05) than when there were fewer (M = 0.86, SD = 0.08), as would be expected.

In Experiment 2, we again observed that mean proportion correct across sessions was significantly lower (paired t(28) = 11.74, p << 0.0001) when total area was controlled (M = 0.79, SD = 0.07) than when item area was controlled (M = 0.87, SD = 0.05). However, in both cases performance was well above chance (lowest t(28) = 22.30, highest p << 0.0001), again demonstrating that area could not have been the only basis for discrimination. We also found that there was a significant parametric effect of numerical ratio when total area was controlled (t(127) = 5.47, p << 0.0001) and when item area was controlled (t(127) = 8.18, p << 0.0001). Finally, we also found that performance on the color task, although well above chance in both cases, was greater (paired t(28) = 2.53, p = 0.02) when more illuminated pixels (M = 0.89, SD = 0.05) were on the screen than when fewer were (M = 0.88, SD = 0.07), as would be expected.

### Testing robustness with a bootstrap analysis

To determine the robustness of our findings and test our power with fewer injection sessions, we performed a bootstrap analysis on our three most important findings: drug-by-side interaction effect on RT in VIP, drug-by-side interaction effect on RT in LIP, and any effect of drug on accuracy in LIP. We resampled our sessions 100 times with replacement. We varied to total number of sessions in each bootstrap sample testing our power at 8, 14, 22, and 28 sessions per experiment. We found our results were robust, with at least 85% power even with as few as 8 sessions (Table 4).

**Table 4:** Effects were robust and apparent with fewer experimental sessions.

Number of sessions per bootstrap	8	14	22	28
Exp 1. VIP muscimol x side interaction on RT	85	85	92	98
Exp 2. LIP muscimol x side interaction on RT	88	96	98	100
Exp 2. LIP any muscimol interaction effect on accuracy	98	100	100	100

We repeated three statistical tests for our main findings. We compared a model of RT including drug x side interactions to one without them for both VIP and LIP, and we compared a model of accuracy including any main effect of drug or interaction with drug to one without for LIP. Numbers indicate the number of bootstraps (out of 100) in which we found an effect at p < 0.05.

## Discussion

The IPS has long been understood to be a critical processing point for the extraction of number from visual scenes [22]. LIP and VIP are thought to be of particular importance for computing ordinal and cardinal numerosity respectively [24,28]. We used reversible inactivation to test the hypothesis that LIP and VIP play a unique role in numerical comparison over and above any role they may play in a control color comparison task. We found that inactivation of VIP and LIP impaired numerical and color comparisons similarly when performed in the contralateral hemifield. VIP inactivation impaired RT in both tasks. LIP inactivation caused delays in RTs as well as a drop in accuracy in both tasks. We found some evidence that accuracy on the color task was selectively impaired following LIP inactivation, however, the effect was small and only emerged in the post-hoc tests, not the GLM results. Our findings do not support a unique causal role for IPS in numerical comparison over and above its role in color comparison.

Because both tasks were approximately equally impaired, it is difficult to ascertain the scope of the deficits caused by IPS inactivation. The number and color tasks rely on many overlapping mechanisms ranging from visual transduction, to basic perceptual integration, to integrative decision-making, to the oculomotor descending pathway, to the contraction of eye muscles required for a response. Anything that affected vision or motor responses could have caused similar deficits in both tasks, just as we observed. For this reason, a third control task unaffected by muscimol would have provided a clearer frame of reference for the current finding. For example, finding that animals were capable of memory guided saccades would have firmly demonstrated that the deficits reported here were not the result of a scotoma or oculomotor deficit. However, we do note that neither LIP nor VIP inactivation has been found to produce deficits to memory-guided saccades in the absence of choice between two stimuli [55,56]. Nevertheless, without a third control task our conclusions are necessarily limited.

It is still worthwhile to consider the pattern of behavioral results and consider the likelihood of different types of underlying deficits caused by IPS inactivation. For example, the observed effects are not likely to be the result of gross impairments in vision or moving the eyes. Even in the muscimol condition, monkeys performed both the number and color tasks well above chance, demonstrating some degree of perceptual integration. Further, inappropriate responses (excluded from the GLM and post-hoc analyses), in which the monkey failed to make an on-target saccade to one of the choice stimuli, comprised less than 6% of all trials in both experiments and were roughly equal in the muscimol and saline conditions (see *Behavioral Tasks* in Methods section). Thus, monkeys were capable of detecting and orienting to targets in the contralateral hemifield after muscimol administration, and the deficits reported here cannot be explained by a simple visual scotoma, complete visual hemi-neglect, or an inability to shift gaze to targets contralateral to the injection site.

Even if we can rule out a gross impairment, we must consider that IPS inactivation caused a less significant deficit to the mechanisms governing orienting responses. Such a deficit can explain the pattern of results we observed following VIP inactivation, which caused a uniform slowing of RT that had no significant interaction with any task variable. Thus, VIP inactivation may have simply resulted in a slowing of saccadic response time to the contralateral hemifield and had no effect on any attention, decision making or information integration process. A potential follow-up experiment could test this possibility by comparing the slowing caused by VIP inactivation in the number and color tasks with RT in a simple memory guided saccade task.

Most studies of VIP outside the numerical cognition literature have focused on visual motion processing. VIP receives strong inputs from the middle temporal (MT) and medial superior temporal (MST) motion areas [57,58]. VIP responds to simple spots of light moving in a preferred direction as well as complex motion generated by an object moving toward or away from the face or head [52]. VIP cells have multimodal coordinated receptive fields whereby some cells that respond to optic flow towards a point on an animal’s face also have tactile receptive fields that respond to stroking the face in the same direction [59]. VIP cells also have coordinated vestibular response fields that encode acceleration in the same direction as a cell’s preferred visual motion direction [60]. The connection between VIP’s role in encoding complex and idiothetic motion and numerical cognition remains unknown.

While a global impairment may explain our VIP findings, we believe a cognitive deficit is more likely to explain the pattern of findings following LIP inactivation for two reasons. First, LIP inactivation caused accuracy as well as RT deficits; however, we found no significant increase in trial time-outs or off target saccades, indicating that the monkeys were able to respond appropriately. Thus, accuracy deficits reflected incorrect decisions rather than oculomotor impairments. Second, we found interactions between drug and trial difficulty, suggesting that the perceptual ambiguity of the to-be-discriminated stimuli was affected by the drug. This is consistent with a large body of literature detailing the close relationship among LIP activity, attention and perceptual decision making.

Although LIP cells fire prior to saccade initiation and respond to the onset of simple static visual stimuli, their responses cannot be described as simply motor or visual [51]. Visual responses in LIP reflect the allocation of spatial attention [36] and the expected value of saccades [37]. Inactivation of LIP does not cause delays in memory guided saccades, but does slow response time on a covert visual search task, consistent with a role in the allocation of spatial attention and in perceptual discrimination [55,56]. LIP neurons also ramp up their activity as perceptual evidence favoring a specific gaze shift accumulates [38]. While this finding can be interpreted as a motor signal and thus that LIP activity does reflect saccade planning, similar accumulation of evidence has been observed in the firing rates of LIP neurons even when motor planning is experimentally dissociated from the decision process [61] and when the behaviorally relevant categories are arbitrarily manipulated, thus demonstrating that LIP neurons encode behaviorally relevant category boundaries independent of spatial attention or motor planning [62,63].

It is possible that, in monkeys, number neurons reported in LIP are part of a more generic system that encodes behaviorally relevant categorical distinctions. This generalized system for perceptual decision making may discriminate dot-motion, number, color, reward size, or any other behaviorally relevant perceptual feature that affects the expected value of a motor action. Indeed, color has been found to be encoded by LIP neurons when it is behaviorally relevant for directing eye movements, but not when it is behaviorally irrelevant [61,64]. Color is also encoded in LIP when it is behaviorally relevant for an upcoming action, but before motor planning can begin, indicating a more abstract role in decision making [65]. Thus, when we inactivated LIP, we may have degraded an animal’s ability to perform any such discrimination. In support of this hypothesis, recent evidence suggests that some posterior parietal number signals observed in human functional imaging experiments are the result of comparison processes, and do not encode numerical magnitude [66]. Against this view is the finding that, although LIP activity is closely correlated with decision making variables, it may not play a causal role in such decisions [67].

The evidence supporting a general-purpose role for LIP in decision making and attention is compelling. However, there is also strong evidence for a unique role of IPS in numerical cognition that must be reconciled with the results of our experiment. The strongest evidence for unique numerical processing in macaque parietal cortex comes from Sawamura, Shima and Tanji [68]. They demonstrated that muscimol inactivation of parietal area 5 selectively disrupted a numerical production task, but not a control task involving the same motor actions. However, it is not clear that the type of production task used in their experiment, in which an animal is trained to produce a number of sequential movements, uses the same cognitive mechanisms as a numerical perception task, in which an animal is trained to discriminate stimuli based on numerosity. The link between these types of tasks is tentative, and they might not rely on the same “number sense” process.

The evidence for a unique role for IPS in numerical cognition is best supported by research in humans. Naturally occurring lesions sometimes result in a condition known as acalculia, a deficit of mathematical cognition. Although usually comorbid with other symptoms in Gerstmann’s syndrome [69], there are examples of “pure” acalculia. In addition to supporting a dissociation of mathematical deficits from other cognitive impairments, the lesion literature also supports the dissociation of rote retrieval of mathematical facts and quantitative abilities within mathematics, with quantitative deficits being specifically associated with IPS lesions [70–73]. Disruption of parietal areas by TMS has been found to impair arithmetic, although the specificity of those effects was not tested [74,75].

Of course, a specific deficit to symbolic mathematics is not the same as a specific deficit to approximate numerical discrimination. While symbolic mathematics is a uniquely human activity, monkeys are capable of more complex numerical manipulation than the comparison task used here. For example, monkeys can associate symbols with numerical quantities [76,77], add and subtract non-symbolic quantities [78], and compare numerical ratios [79]. Future research should explore whether IPS shows greater specificity for these more demanding numerical processes.

Considering the literature showing IPS does play a significant and unique role in numerical cognition and given the danger of drawing conclusions from negative results, we also need to consider the possibility that our study was underpowered. We did demonstrate significant deficits in the color task, ruling out the possibility that IPS plays no role in color discrimination. However, given the role for LIP in attention and decision-making outlined above, this was an unlikely possibility. It is still possible that IPS plays a slightly more important role in numerical discrimination than in color discrimination, and that with more sessions we may have observed significantly greater deficits in the number task. Against this possibility is our post-hoc analysis showing that accuracy deficits following LIP inactivation were actually greater in the color condition than in the number condition. Thus, it is unlikely that more sessions would show a significant effect in the opposite direction.

In addition to VIP and LIP, numerosity sensitive neurons have also been found in dorsolateral prefrontal cortex (DLPFC) [24]. It may be that IPS and DLPFC form a network of number processing areas and that concurrent inactivation of DLPFC may have caused a more notable decrement in discrimination performance. However, it is unclear how much specificity such a network could have for numerical cognition; it is unlikely that DLPFC inactivation would cause specifically numerical deficits given its involvement in many learned categorical discriminations [80–82].

It is noteworthy that, while our analyses confirm that monkeys used number to perform the number task, their performance was enhanced when the total area of the array items was congruent with number. On these trials the total number of red dots and the total area of red dots were both greater in the same array, and the performance increase on these trials indicates that monkeys were partially biased by area when making numerical discriminations. Similar biases are observed in humans making numerical discriminations [53,54]. One possible explanation of the decrement in the number task is that IPS inactivation prevented monkeys from dissociating number and area; another possibility is that inactivation prevented monkeys from successfully using area as a proxy for number on congruent trials. However, area effects alone cannot explain the deficits we observed in the color task, in which area was always equated between the arrays.

More generally, the results of these experiments are consistent with the idea that the IPS is specialized for the perception and comparison of various magnitudes, such as time, space, and size, as well as numerosity and area [83–86]. Endorsing this view, functional imaging studies in humans demonstrate that comparing different magnitudes (such as spatial location, luminance, size and duration) activates overlapping areas in the intraparietal sulcus [87–91]. Single cell recordings in monkeys have demonstrated that both numerosity and line length are encoded in IPS in partially overlapping populations of neurons [25]. Disruption of IPS by TMS in humans affects number, line length, size, and shape judgments [31,92,93] and affects the congruency effect of size on number comparison [35,94]. Therefore, it is possible that monkeys in our study perceived color along an ordinal dimension of “reddishness” and that the color task engaged IPS “ordinality” circuits in common with the numerical estimation task. This hypothesis could be tested by comparing tasks in which stimuli existing on a continuum must be discriminated, such as our number and color tasks, with a task involving clear categorical discriminations.

## Supporting information

S1 Table Parameter estimates for the full models in Experiment 1 (VIP injections). (PDF)

S2 Table Parameter estimates for the full models in Experiment 2 (LIP injections). (PDF)
