# Supplementary material for: Pharmacological inactivation does not support a unique causal role for intraparietal sulcus in the discrimination of visual number
Source: PLoS One. 2017 Dec 14;12(12):e0188820. doi: 10.1371/journal.pone.0188820 (PMC5730202; doi:10.1371/journal.pone.0188820)
Supplement: S1 Table — (PDF) [file pone.0188820.s001.pdf]

	Exp 1: VIP Acc		Exp 1: VIP RT	
Coefficient Name	Estimate (SE)	p Value	Estimate (SE)	p Value
(Intercept)	0.2519 (0.4213)	0.5499	-2.7506 (0.0493)	0
Drug	-0.0534 (0.1084)	0.622	0.0721 (0.0698)	0.3018
Task	0.2279 (0.1532)	0.1368	-0.0379 (0.0356)	0.2877
Side	0.0608 (0.1645)	0.7118	0.0986 (0.0371)	0.008
Time	-0.0766 (0.1112)	0.4908	0.0805 (0.0253)	0.0014
NumDifficulty	1.9716 (0.094)	2.51E-97	-0.027 (0.0216)	0.212
HueDifficulty	10.2982 (0.7391)	4.55E-44	-0.3775 (0.14)	0.007
Drug : Task	0.1168 (0.2168)	0.5902	-0.0485 (0.0507)	0.3383
Drug : Side	0.1112 (0.2716)	0.6821	0.0629 (0.0628)	0.3164
Task : Side	0.4512 (0.314)	0.1507	0.1234 (0.0704)	0.0798
Drug : Time	0.1198 (0.1563)	0.4436	-0.009 (0.036)	0.8025
Task : Time	0.1825 (0.2225)	0.4121	0.0191 (0.0505)	0.7055
Side : Time	-0.0826 (0.0945)	0.3821	-0.0596 (0.024)	0.0129
Drug : NumDiff	-0.0631 (0.1329)	0.635	0.028 (0.0308)	0.3631
Side : NumDiff	-0.5443 (0.1862)	0.0035	0.1229 (0.0432)	0.0044
Time : NumDiff	-0.0821 (0.131)	0.5309	-0.077 (0.0304)	0.0113
Drug : HueDifficulty	0.2017 (1.0462)	0.8471	-0.1381 (0.1991)	0.488
Side : HueDifficulty	-0.5537 (1.5451)	0.7201	0.4669 (0.2751)	0.0897
Time : HueDifficulty	1.5581 (1.0957)	0.155	-0.153 (0.1985)	0.4407
Drug : Task : Side	-0.0645 (0.5342)	0.9039	0.0367 (0.1232)	0.7661
Drug : Task : Time	-0.0757 (0.3127)	0.8087	-0.0475 (0.072)	0.5092
Drug : Side : Time	0.1223 (0.3235)	0.7055	0.1117 (0.0755)	0.1393
Drug : Side : NumDiff	-0.2553 (0.3226)	0.4288	0.0944 (0.076)	0.2144
Drug : Time : NumDiff	-0.076 (0.1859)	0.6829	-3.97E-04 (0.0435)	0.9927
Drug : Side : HueDiff	-1.2811 (2.5942)	0.6214	0.1587 (0.4808)	0.7413
Drug : Time : HueDiff	-1.1585 (1.5298)	0.4489	-0.0135 (0.2823)	0.9618
Drug : Task : Side : Time	-0.753 (0.6188)	0.2236	-0.2312 (0.1433)	0.1066
Drug : Side : Time : NumDiff	0.4219 (0.3702)	0.2544	-0.0115 (0.0887)	0.8972
Drug : Side : Time : HueDiff	-1.6653 (3.0045)	0.5794	-0.6048 (0.5572)	0.2777

Table S1. Parameter estimates for the full models in Experiment 1 (VIP injections).
