# Supplementary material for: Pharmacological inactivation does not support a unique causal role for intraparietal sulcus in the discrimination of visual number
Source: PLoS One. 2017 Dec 14;12(12):e0188820. doi: 10.1371/journal.pone.0188820 (PMC5730202; doi:10.1371/journal.pone.0188820)
Supplement: S2 Table — (PDF) [file pone.0188820.s002.pdf]

	Exp 2: LIP Acc		Exp 2: LIP RT	
Coefficient Name	Estimate (SE)	p Value	Estimate (SE)	p Value
(Intercept)	0.3223 (0.3816)	0.3984	-2.7672 (0.0498)	0
Drug	-0.1728 (0.106)	0.1029	0.1127 (0.0676)	0.0957
Task	0.2601 (0.1498)	0.0825	-0.1006 (0.0341)	0.0032
Side	-0.2672 (0.1625)	0.1001	0.0793 (0.0355)	0.0254
Time	-0.2233 (0.11)	0.0423	0.1088 (0.0243)	7.79E-06
NumDifficulty	1.8354 (0.0896)	5.88E-93	-0.0074 (0.0207)	0.7197
HueDifficulty	10.1928 (0.7285)	2.04E-44	-0.4925 (0.1345)	2.51E-04
Drug : Task	-0.1216 (0.2119)	0.5662	0.096 (0.0491)	0.0507
Drug : Side	0.3069 (0.2661)	0.2488	0.2395 (0.0608)	8.18E-05
Task : Side	0.2305 (0.3093)	0.4561	0.0081 (0.0673)	0.9046
Drug : Time	0.2073 (0.1559)	0.1835	0.0203 (0.0351)	0.5629
Task : Time	0.2112 (0.2199)	0.3369	-0.0299 (0.0486)	0.5387
Side : Time	0.1362 (0.0923)	0.1401	-0.0262 (0.0229)	0.2524
Drug : NumDiff	0.2404 (0.1316)	0.0678	-0.0648 (0.0302)	0.0316
Side : NumDiff	-0.0178 (0.1816)	0.9218	0.0384 (0.0412)	0.3513
Time : NumDiff	0.1318 (0.1285)	0.305	-0.1482 (0.0294)	4.58E-07
Drug : HueDifficulty	-0.2338 (1.0207)	0.8188	0.1963 (0.1928)	0.3086
Side : HueDifficulty	1.2889 (1.5363)	0.4015	-0.2345 (0.2644)	0.3752
Time : HueDifficulty	2.2623 (1.0967)	0.0391	-0.4867 (0.1912)	0.0109
Drug : Task : Side	-0.1032 (0.5228)	0.8436	-0.0367 (0.1195)	0.7584
Drug : Task : Time	0.345 (0.3117)	0.2685	0.0434 (0.0701)	0.5353
Drug : Side : Time	0.2523 (0.3193)	0.4294	0.0127 (0.0737)	0.8636
Drug : Side : NumDiff	-0.4493 (0.3267)	0.1691	0.0619 (0.0736)	0.4005
Drug : Time : NumDiff	-0.6849 (0.1832)	1.85E-04	0.0862 (0.0429)	0.0445
Drug : Side : HueDiff	-2.8889 (2.5278)	0.2531	-0.1348 (0.467)	0.7729
Drug : Time : HueDiff	-0.3571 (1.553)	0.8181	0.4124 (0.2747)	0.1333
Drug : Task : Side : Time	-0.8455 (0.6113)	0.1667	0.0887 (0.1402)	0.5269
Drug : Side : Time : NumDiff	-0.1397 (0.3677)	0.7039	-0.058 (0.0877)	0.5083
Drug : Side : Time : HueDiff	-8.1765 (2.9956)	0.0063	0.0812 (0.5469)	0.8819

Table S2. Parameter estimates for the full models in Experiment 2 (LIP injections).
